# Postoperative full leg radiographs exhibit less residual coronal varus deformity compared to intraoperative measurements in robotic arm-assisted total knee arthroplasty with the MAKO™ system

**DOI:** 10.1007/s00167-023-07386-z

**Published:** 2023-03-25

**Authors:** Claudio Glowalla, Severin Langer, Ulrich Lenze, Igor Lazic, Michael T. Hirschmann, Florian Hinterwimmer, Rüdiger von Eisenhart-Rothe, Florian Pohlig

**Affiliations:** 1 Department of Orthopaedic Surgery, Klinikum Rechts der Isar, Technical University Munich, Ismaninger-Strasse 22, 81675 Munich, Germany; 2 BG Unfallklinik Murnau, Professor-Kuentscher-Strasse 8, 82418 Murnau am Staffelsee, Germany; 3 Department of Orthopaedic Surgery and Traumatology, Kantonsspital Baselland (BruderholzLiestalLaufen), 4101 Bruderholz, Switzerland

**Keywords:** Alignment, Total knee arthroplasty, TKA, Computer-aided surgery, CAS, Robotic-assisted TKA, raTKA, Full limb radiograph, FLR, MAKO

## Abstract

**Purpose:**

Robotic arm-assisted total knee arthroplasty (raTKA), currently a major trend in knee arthroplasty, aims to improve the accuracy of implant positioning and limb alignment. However, it is unclear whether and to what extent manual radiographic and navigation measurements with the MAKO™ system correlate. Nonetheless, a high agreement would be crucial to reliably achieve the desired limb alignment.

**Methods:**

Thirty-six consecutive patients with osteoarthritis and a slight-to-moderate varus deformity undergoing raTKA were prospectively included in this study. Prior to surgery and at follow-up, a full leg radiograph (FLR) under weight-bearing conditions was performed. In addition, a computed tomography (CT) scan was conducted for preoperative planning. The hip–knee–ankle angle (HKA), mechanical lateral distal femur angle (mLDFA), mechanical medial proximal tibial angle (mMPTA) and joint line convergence angle (JLCA) were measured in the preoperative and follow-up FLR as well as in the CT scout (without weight-bearing) by three independent raters. Furthermore, the HKA was intraoperatively assessed with the MAKO™ system before and after raTKA.

**Results:**

Significantly higher HKA values were identified for intraoperative deformity assessment using the MAKO system compared to the preoperative FLR and CT scouts (*p* = 0.006; *p* = 0.05). Intraoperative assessment of the HKA with final implants showed a mean residual varus deformity of 3.2° ± 1.9°, whereas a significantly lower residual varus deformity of 1.4° ± 1.9° was identified in the postoperative FLR (*p* < 0.001). The mMPTA was significantly higher in the preoperative FLR than in the CT scouts (*p* < 0.001). Intraoperatively, the mMPTA was adjusted to a mean of 87.5° ± 0.9° with final implants, while significantly higher values were measured in postoperative FLRs (*p* < 0.001). Concerning the mLDFA, no significant differences could be identified.

**Conclusion:**

The clinical importance of this study lies in the finding that there is a difference between residual varus deformity measured intraoperatively with the MAKO™ system and those measured in postoperative FLRs. This has implications for preoperative planning as well as intraoperative fine-tuning of the implant position during raTKA to avoid overcorrection of knees with slight-to-moderate varus osteoarthritis.

**Level of evidence:**

Level IV.

## Introduction

Robotic arm-assisted total knee arthroplasty (raTKA), currently a major trend in knee arthroplasty, aims to improve the accuracy of implant positioning and limb alignment to improve clinical outcomes as well as implant survival [1, 8, 25, 32].

MAKO™ total knee robotic arm-assisted surgery (Stryker, Kalamazoo, USA) is one of the most popular and widespread systems allowing the surgeon to perform intraoperative quantitative ligament balancing, to adjust the implant position and to “semiactively” interact with the robotic arm during bone preparation. The procedure is based on a preoperative computed tomography (CT) scan of the patient’s lower limb and a three-dimensional planning of the implant size and orientation as well as the limb alignment.

This technique achieves a high accuracy of femoral and tibial bone resection with a difference between planned and actual cuts of less than 0.5 mm [20]. Furthermore, a high accuracy of coronal limb alignment has been reported comparing planned data with intraoperative values with final implants as well as postoperative CT scans [20, 21]. However, the coronal limb alignment was measured in a lying and unloaded condition in the CT scans as well as during intraoperative assessments, which may exhibit different results compared to those under weight-bearing conditions. This could lead to a mismatch in coronal limb alignment between the intraoperative assessment and the postoperative outcome [3, 18, 31].

Despite potential differences, no in vivo studies have focused on the pre-, intra- and postoperative analysis of coronal alignment in raTKA with the MAKO® system under weight-bearing and non-weight-bearing conditions. Yet, this knowledge is crucial for intraoperative adjustment of implant orientation and alignment. Therefore, the present prospective study was conducted to address the following hypotheses: (1) Preoperative radiographic coronal alignment under weight-bearing conditions is significantly different from preoperative non-weight-bearing radiographic alignment and intraoperative deformity assessment, and (2) postoperative radiographic coronal alignment measurements exhibit less residual varus deformity than intraoperative measurements with the MAKO™ robotic system with final implants.

## Materials and methods

All patients were provided with all relevant information before the beginning of the study, and written consent was obtained. This study was approved by the institutional ethics committee of the Technical University Munich (IRB number 409/20S).

**Fig. 1:**
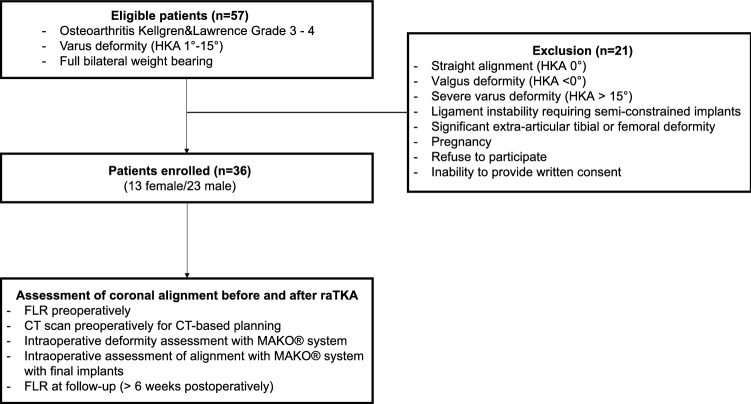
Flowchart of patient inclusion

Thirty-six consecutive cases with osteoarthritis and a slight-to-moderate varus deformity were prospectively included in this study (Fig. 1). Demographic data of the subjects are summarised in Table 1. Patients with severe deformities potentially requiring semiconstrained or constrained implants were excluded. In all cases, raTKAs were performed using the MAKO™ system (Stryker, Kalamazoo, USA) and the Triathlon Total Knee system. All surgeries were executed by two senior knee arthroplasty surgeons who had undergone intensive training on the technique and had performed more than 100 raTKA cases prior to this study [19]. Prior to surgery, a full leg radiograph (FLR) under weight-bearing conditions was performed in addition to the CT scan required for preoperative planning. The initial 3D planning was preoperatively adjusted by the surgeon regarding implant position and size according to a restricted kinematic alignment philosophy.

**Table 1:** Demographic data

Variable	
Sex, *n* (%)	36 (100)
Female	13 (36)
Male	23 (64)
Age (years; SD)	69.5 (8.6)
BMI (SD)	27.8 (4.5)
Mean postoperative follow-up (weeks; SD)	6.9 (2.6)

Total knee arthroplasty (TKA) was performed using a medial parapatellar approach, and posterior stabilised implants were used in all cases. After positioning of the femoral and tibial navigation arrays as well as the checkpoints, initial bone mapping was performed. Then, baseline data regarding joint gaps and alignment were dynamically assessed. Subsequently, after the removal of all relevant osteophytes, gap balancing was performed with spacers of different heights, and the implant position was modified accordingly to achieve equal medial and lateral gap widths in full extension and 90° of flexion. Target coronal alignment and equal joint gap widths were verified with trial implants and recorded after definitive implantation.

The hip–knee–ankle angle (HKA), mechanical lateral distal femur angle (mLDFA), mechanical medial proximal tibia angle (mMPTA) and joint line convergence angle (JLCA) were measured twice for each case in the preoperative full limb radiograph (FLR), in the CT scout (without weight-bearing) and in the postoperative FLR (6–8 weeks postoperatively) by 3 independent senior arthroplasty surgeons. HKA was defined as the acute angle formed by the mechanical femoral axis (centre of the femoral head to centre of the femoral condyles) and the mechanical tibial axis (centre of the tibial plateau to centre of the talus) as previously described [13, 16, 31]. Positive values were considered as a varus and negative values as a valgus deformity. mLDFA was defined as the lateral angle between the mechanical femoral axis, as described above, and a tangential line between the most distal points of the medial and lateral femoral condyle. mMPTA was measured between the mechanical tibial axis and a tangential line between the deepest points of the medial and lateral tibial plateau. JLCA was formed by the tangential lines between the most distal points of the medial and lateral femoral and between the deepest points of the medial and lateral tibial plateau.

Data under weight-bearing conditions (pre- and postoperative long leg radiographs) were compared with the measurement in the CT scouts and intraoperative values without weight-bearing.

### Statistical analysis

All the data collected in this study were recorded and analysed using SPSS 26 software (IBM, Armonk, NY). The normality of the data was assessed by the Shapiro‒Wilk test. Means and standard deviations were calculated and compared using a t-test for paired samples. Inter- and intrarater reliability was determined by the intraclass correlation coefficient and Pearson’s correlation coefficient, respectively.

A sample-size power analysis with *β* = 0.20 and *α* = 0.05 was performed for a pilot study using the mean difference in the HKA in loaded and unloaded conditions of 2° as previously published by Paternostre et al. for high degrees of varus osteoarthritis [16]. Based on this analysis, a minimum of 36 subjects were needed to power the study adequately.

## Results

The overall range of motion (ROM), extension and flexion were significantly improved at follow-up compared to preoperative values (Table 2). The mean HKA, mMPTA, mLDFA and JLCA in the preoperative FLR, CT scouts, intraoperative measurements and postoperative FLR are summarised in Table 3.

**Table 2:** Range of motion preoperatively and at the postoperative follow-up visit; *p* ≤ 0.05 indicates statistical significance

	Preoperative assessment	Postoperative follow-up	*P* value
Range of motion (°; SD)			
Extension	4.7 (6.3)	1.7 (3.2)	0.007
Flexion	117.9 (13.6)	121.4 (8.9)	0.027
ROM total	113.2 (17.2)	119.7 (10.3)	0.002

**Table 3:** Pre-, intra- and postoperative mechanical angles; pre- and postoperative measurements were performed manually, and intraoperative values were assessed with the MAKO™ robotic system

	Preoperative FLR (weight-bearing)	Preoperative CT scout (non-weight-bearing)	Intraoperative deformity (MAKO™-system; non-weight-bearing)	Intraoperative with final implants (MAKO™-system; non-weight-bearing)	Postoperative FLR (weight-bearing)
HKA (°; SD)	6.0 (3.4)	6.4 (3.5)	7.3 (3.3)	3.2 (1.9)	1.4 (1.9)
mMPTA (°; SD)	86.6 (2.5)	85.2 (2.6)	–	87.5 (0.9)	88.7 (1.3)
mLDFA (°; SD)	88.9 (2.1)	88.8 (2.0)	–	89.8 (1.1)	89.9 (1.3)
JLCA (°; SD)	3.7 (1.2)	3.0 (1.3)	–	–	− 0.2 (0.6)

A significant difference in the HKA was identified for intraoperative deformity assessment using the MAKO system compared to the HKA values in the preoperative FLR and the CT scouts (*p* = 0.006; *p* = 0.05). Intraoperative assessment of the HKA with final implants showed a mean residual varus deformity of 3.2° ± 1.9°, whereas a significantly lower residual varus deformity of 1.4° ± 1.9° was identified in the postoperative FLR (p < 0.001). The effect size according to Cohen was small regarding the difference in the HKA in the preoperative FLR and intraoperative deformity assessment (*d* = 0.3) but large for the difference between the intraoperative and postoperative residual deformity (*d* = 0.9).

A significantly higher mMPTA was found in the preoperative FLR compared to the CT scouts (*p* < 0.001). The mMPTA was intraoperatively adjusted to a mean of 87.5° ± 0.9° with final implants. However, a significantly higher mMPTA was identified in the postoperative FLR (*p* < 0.001).

No significant difference regarding the mLDFA could be identified, either between preoperative FLRs and CT scouts or between intraoperative coronal femoral implant positioning and postoperative radiographs.

The JLCA was significantly different in preoperative FLRs (3.8° ± 1.2°) and CT scouts (3.1° ± 1.3°; *p* < 0.001) with a moderate to large effect size (*d* = 0.7). Furthermore, a significant difference could be identified between preoperative values in FLRs and CT scouts compared to the JLCA in postoperative FLRs (*p* < 0.001).

Overall, an excellent inter- and intrarater reliability was found (Table 4).

**Table 4:** Intrarater reliability (Pearson’s correlation coefficient) and interrater reliability (intraclass correlation coefficient) for all manual measurements

	Intrarater reliability			Interrater reliability
	Rater 1	Rater 2	Rater 3	
HKA				
FLR (preop)	0.98	0.98	0.99	0.96
CT scout (preop)	0.98	0.98	0.99	0.99
FLR (postop)	0.98	0.95	0.99	0.86
mMPTA				
FLR (preop)	0.92	0.96	0.98	0.95
CT scout (preop)	0.88	0.95	0.98	0.97
FLR (postop)	0.86	0.90	0.78	0.89
mLDFA				
FLR (preop)	0.88	0.95	0.99	0.95
CT scout (preop)	0.88	0.96	0.95	0.91
FLR (postop)	0.90	0.86	0.95	0.70
JLCA				
FLR (preop)	0.65	0.93	0.92	0.81
CT scout (preop)	0.71	0.93	0.69	0.79
FLR (postop)	0.73	0.93	0.91	0.41

## Discussion

The most important finding of this study was that residual varus deformity after raTKA was significantly lower in the postoperative full weight-bearing FLR than in the intraoperative assessment with final implants. Furthermore, more varus deformity was preoperatively measured under non-weight-bearing conditions and during intraoperative assessments compared to the preoperative radiographic coronal alignment under weight-bearing conditions.

Good to excellent intra- and interobserver reliability for all measurements of the HKA, mMPTA and mLDFA was found in agreement with other previously published studies [4, 11, 30].

Measurement of HKA in FLRs is still the gold standard to plan, perform, and evaluate the alignment of TKA [18, 24]. However, it has previously been shown that various factors influence radiographic measurements of coronal limb alignment. Among these, compared with full extension, flexion contracture of the knee joint is considered to give more semblance of coronal valgus in weight-bearing FLRs [12]. However, Krackow et al. demonstrated that knee flexion up to 10° only exhibits a small effect on the HKA measured in FLRs [9]. Furthermore, internal or external rotation of the knee joint during the FLR may additionally influence the HKA measurement, as previously shown by Lonner et al. [12]. The authors demonstrated that higher valgus angles were measured in radiographs with internally rotated knee joints compared to external rotation. In contrast, Kawakami et al. showed an increased influence of knee rotation on radiographic mechanical limb alignment with increased knee flexion [7]. On the other hand, considering a mean postoperative flexion contracture of only 1.7° and the use of a highly standardised protocol for FLRs in the present study, flexion contracture and radiographic malrotation seem to be unlikely reasons for the present postoperative findings.

However, slightly more varus was observed in the CT scouts without weight-bearing compared to FLRs despite various studies suggesting different results [16, 23, 29]. In this context, Paternostre et al. identified an increase in varus deformity of 2° or more under weight-bearing conditions when comparing the coronal alignment of osteoarthritic knee joints in FLR and MRI scans [16]. Similar results were published by Specogna et al. and Winter et al. [23, 29]. In the present study, the CT scans were performed in supine position without a leg holder. In this position, the leg usually exhibits a slight external rotation of the hip joint which also leads to a certain degree of external rotation of the knee. It could be hypothesised that this external rotation may give more semblance of varus compared to neutral position [7, 12]. Additionally, the weight of the leg in the supine position may improve a potential flexion contracture. Consequently, the measurement of coronal alignment could reflect higher values of varus deformity than that measured in FLRs with more flexion contracture and neutral rotation.

The present differences in the HKA measured by the MAKO™ system and in FLRs may also be attributed to the extent of weight-bearing. Despite previous studies suggesting no significant influence of weight-bearing on coronal alignment [5, 22], Yaffe et al. identified a tendency towards a higher difference in HKA values measured by a computer-aided surgery system (CAS) compared to FLRs in patients with higher degrees of preoperative limb deformity [30]. Similarly, Zahn et al. reported about significantly more valgus alignment at 10 days compared to 3 months after conventional TKA aiming at neutral mechanical alignment [31]. The authors identified full weight-bearing as the main factor for the change in coronal alignment. However, considering the mean follow-up of 6.9 weeks in this study, the full weight-bearing ability could be assumed.

In this study, more varus deformity was found intraoperatively than with preoperative weight-bearing FLRs. In contrast, Wang et al. identified more varus in preoperative full weight-bearing radiographs than in intraoperative assessments in supine position [27]. In their cohort, however, the severity of osteoarthritis was less severe than that in the present study, potentially explaining the discrepancy. On the other hand, Wilcox et al. reported similar results in their cohort with advanced osteoarthritis treated by TKA [28]. Similarly, Barbotte et al. identified an overestimation of the deformity in FLRs compared to values measured by a CAS system [3]. Explaining the present results, it could be hypothesised that despite a mean preoperative flexion contracture of only 4.7°, anaesthesia and muscular relaxation may lead to full extension intraoperatively and, thus, reveal the full extent of varus deformity.

In a comparative study regarding coronal alignment of osteoarthritic knee joints with varus deformity undergoing navigated medial high tibial open-wedge osteotomy, Wang et al. found more valgus postoperatively under weight-bearing conditions than intraoperatively measured by a navigation system in supine position [27]. Accordingly, Schoenmakers et al. demonstrated a mean difference of 1.5° between intraoperative coronal alignment with final implants assessed by a navigation system and postoperative measurement of HKA in weight-bearing FLR [18]. These data support the postoperative results of the present study. Explaining these findings, medial collateral instability could be assumed. However, a symmetrical joint gap width was intraoperatively verified with final implants. Furthermore, a mean JLCA of − 0.2° was measured in the postoperative long leg radiographs, making instability unlikely to account for the present findings.

The mean mMPTA was intraoperatively adjusted to 87.5 ± 0.9 despite lower values in the preoperative measurements. Intraoperative implant positioning was based on equal medial and lateral bony resection heights, if possible. However, a certain degree of medial tibial bone defects in severe varus osteoarthritis must be taken into account. Furthermore, the principle of restricted kinematic alignment, as previously published by several authors, was pursued in the present study, limiting the combined coronal femoral and tibial implant positioning to 3 degrees [10, 17, 26]. Interestingly, significantly less varus positioning of the tibial component was measured in the postoperative long leg radiograph. This could be explained by an internal rotation of the knee joint during FLR as previously proposed by Lonner et al. [12].

Regarding the femoral implant position, a mean mLDFA of approximately 89° preoperatively and approximately 90° intra- and postoperatively was observed without any significant difference. These results are in accordance with several previous studies proposing that the causative factor for varus deformity in knee osteoarthritis is often a bony defect of the medial tibial plateau, a pathologically decreased mMPTA or a combination of both [2, 6]. In contrast, the distal femoral bone configuration is within physiological values in most cases only requiring femoral resurfacing without distinct reorientation of the femoral component [26]. Overall, highly accurate implant positioning without significant outliers could be achieved with the MAKO™ CAS system, confirming previously published results [8, 14, 15].

However, several limitations of this study must be noted. First, the relatively small number of subjects included in this pilot study could have influenced the present results. However, it was the aim to investigate a rather homogenous cohort of varus deformed osteoarthritis knees. Second, subjects were not subdivided into different study groups according to the degree of osteoarthritis and the extent of preoperative deformity. This may conceal potential differences. Third, only one CAS system was used and compared to values obtained from FLRs in a single centre. Thus, a systematic error related to the CAS system or the technique of FLR acquisition cannot be excluded. Fourth, measurement of the mechanical angles was manually performed by FLR. Despite a high inter- and intrarater reliability, a certain degree of error could have influenced the present results.

The importance of this study for daily clinical practice lies in the finding that more residual varus deformity was measured during intraoperative assessment with final implants compared to postoperative FLRs. Thus, care should be taken during intraoperative alignment to avoid unintended overcorrection of knees with preoperative varus deformity with potentially unfavourable results.

## Conclusion

In this study, more residual varus deformity was measured intraoperatively compared to postoperative FLRs. Despite a lack of knowledge on how to clinically interpret the present discrepancy in alignment, care should be taken to avoid clinically relevant overcorrection of knees with varus deformity osteoarthritis.

## Data Availability

All data related to this study are contained within the manuscript.

## Abbreviations

raTKA: Robotic arm-assisted total knee arthroplasty
CT: Computed tomography
FLR: Full leg radiograph
TKA: Total knee arthroplasty
HKA: Hip–knee–ankle angle
mLDFA: Mechanical lateral distal femur angle
mMPTA: Mechanical medial proximal tibia angle
JLCA: Joint line convergence angle
CAS: Computer-aided surgery
